# Dysbiosis of the Upper Gastrointestinal Tract in Head-and-Neck Cancer Survivors: A Pilot Study Using the Capsule Sponge Device

**DOI:** 10.3390/cancers16203528

**Published:** 2024-10-18

**Authors:** Natalia Zeber-Lubecka, Maria Kulecka, Michalina Dabrowska, Anna Kluska, Magdalena Piątkowska, Maryla Helena Turkot, Nastazja Dagny Pilonis, Aisha Yusuf, Karol Nowicki-Osuch, Michal Mikula, Jerzy Ostrowski

**Affiliations:** 1 Department of Gastroenterology, Hepatology and Clinical Oncology, Centre of Postgraduate Medical Education, 02-781 Warsaw, Poland; natalia.zeber-lubecka@cmkp.edu.pl (N.Z.-L.); maria.kulecka@cmkp.edu.pl (M.K.); maryla.turkot@nio.gov.pl (M.H.T.); nastazja.pilonis@nio.gov.pl (N.D.P.); 2 Department of Genetics, Maria Sklodowska-Curie National Research Institute of Oncology, 02-781 Warsaw, Poland; michalina.dabrowska@nio.gov.pl (M.D.); anna.kluska@nio.gov.pl (A.K.); magdalena.piatkowska@nio.gov.pl (M.P.); michal.mikula@nio.gov.pl (M.M.); 3 Department of Cancer Prevention, Maria Sklodowska-Curie National Research Institute of Oncology, 02-781 Warsaw, Poland; 4 Department of Gastrointestinal Oncology, Maria Sklodowska-Curie National Research Institute of Oncology, 02-781 Warsaw, Poland; 5 Early Cancer Institute, Department of Oncology, University of Cambridge, Cambridge CB2 1TN, UK; ay327@cam.ac.uk; 6 German Cancer Research Center (DKFZ), 69120 Heidelberg, Germany

**Keywords:** capsule-sponge device, microbial dysbiosis, head and neck squamous cell cancer (HNSCC), esophageal squamous intraepithelial neoplasia, early esophageal squamous cell carcinoma (ESCC)

## Abstract

Background: A non-endoscopic capsule-sponge device allows sampling the entire length of the esophagus. Here, we compared microbiomes of the oral cavity, esophagus, and gastric corpus collected by oral swab, capsule-sponge device, and endoscopic biopsy, respectively, in patients representing three distinct risk profiles for esophageal squamous cell carcinoma (ESCC). Methods: The study enrolled 11 patients with esophageal squamous intraepithelial neoplasia, 21 patients after curative treatment for head and neck squamous cell cancer (HNSCC) (HNSCC survivors), and 40 patients with functional dyspeptic (FD) symptoms. Microbial genomic DNA was analyzed using 16S rRNA gene amplicon sequencing. Results: The Shannon index of the capsule-sponge sample microbiota was significantly higher in FD group than in patients after treatment for HNSCC, and the Chao index of gastric samples differed between HNSCC survivors and FD patients. Analysis of the β-diversity of FD patients, HNSCC, and esophageal squamous intraepithelial neoplasia showed that different genera formed at each location. The abundance of 205, 116, and 9 genera differed between FD patients and HNSCC survivors in the gastric, capsule-sponge, and oral samples, respectively; 33 genera differed between the FD group and patients with esophageal squamous intraepithelial neoplasia in capsule-sponge samples. Conclusions: The bacterial communities of the upper digestive tract were clustered according to the anatomic site. Despite substantial differences in gastric and esophageal microbiota samples between FD patients and HNSCC survivors, the microbial members and diversity showed small differences between FD patients and those with esophageal squamous intraepithelial neoplasia. It remains unclear whether gastric and esophageal dysbiosis is associated with or is a consequence of treatment for HNSCC.

## Simple Summary

This study explores the microbiome of the upper digestive tract using a non-endoscopic capsule-sponge device, aiming to understand the differences in microbial communities among patients with varying risks for esophageal squamous cell carcinoma (ESCC). We compared microbiomes from the oral cavity, esophagus, and stomach across three groups: patients with esophageal squamous intraepithelial neoplasia, head and neck squamous cell cancer (HNSCC) survivors, and patients with functional dyspepsia. By analyzing microbial DNA using 16S rRNA gene sequencing, the study found distinct differences in microbiota diversity and composition between FD patients and HNSCC survivors, particularly in gastric and esophageal samples. However, differences between FD patients and those with esophageal neoplasia were minimal. These findings could help identify microbial patterns associated with ESCC risk and guide future research on the role of microbiome alterations in cancer development, potentially leading to new diagnostic or therapeutic approaches.

## 1. Introduction

Microbial communities naturally inhabit the human body with widespread involvement in physiological, metabolic, and immunological processes. Although the composition and function of microbiota located in different sites may depend on specific environmental conditions that give rise to regional specificity in microbial populations [1], most studies so far have focused on the gut microbiota. In contrast, the microbiota of the upper gastrointestinal tract has attracted less attention [2]. The gastroesophageal microbiota is shaped by the oral cavity, pharynx and stomach due to the migration of oral bacteria to the esophagus and the reflux of the microflora in the stomach [3]. In addition to anatomic location, age, diet, use of proton pump inhibitors (PPIs), oral hygiene and smoke can result in esophageal microbiota dysbiosis.

Culture-independent molecular techniques suggest that the oral, pharyngeal, esophageal, and gastric microbiota can be clustered according to anatomic site [4]; the microbial communities of the gastric body are distinct from those of the esophagus, and the communities of the stomach and esophagus differ from those in the mouth and the lower GI tract [4].

The microbiota in the upper digestive tract may play an important role in the development of gastric and esophageal cancer, and the difference in esophageal microbiome composition between healthy individuals and those with cancerous or cancer-predisposing conditions is well established. A cross-sectional study on a Chinese cohort first showed an increase in Gram-negative bacteria linked to adenocarcinoma and Barrett’s esophagus. Research by Liu et al. [5] revealed distinct bacterial communities among normal esophagus, reflux esophagitis, and Barrett’s esophagus in Japanese patients, with Streptococcus prevalent in normal and reflux esophagitis patients, while *Veillonella* dominated in Barrett’s esophagus. Additionally, *Fusobacterium* was present in patients but absent in healthy esophageal tissue. Another study found that *Streptococcus* and *Prevotella* were dominant and significantly associated with Barrett’s esophagus.

Although the oral cavity has a higher bacterial abundance than the esophagus [6], its microbial residents, dominated by *Streptococcus*, *Veillonella*, *Neisseria*, *Haemophilus*, and *Prevotella* species, play an important role in the seeding of downstream sites of the GI tract [7,8,9,10,11,12,13]. Changes in the microbiota of the lower esophagus have been reported in gastro-esophageal reflux disease, Barrett’s esophagus (BE), eosinophilic esophagitis, and esophageal carcinoma [14]. The esophageal microbiota of healthy individuals is dominated by streptococci species, and its composition differs between subjects with a normal esophagus and those with reflux esophagitis and BE by the increased abundance of *Prevotella*, *Veillonella*, *Haemophilus*, *Neisseria*, *Rothia*, and *Fusobacterium* [4,13,15,16,17,18,19]. However, another study [19] failed to detect such reflux-associated differences.

The microbial density within the stomach ranges from 10^2^ to 10^4^ CFU/g, depending on the pH of the gastric lumen [20]. The gastric microbiome comprises Gram-negative and Gram-positive commensal bacteria, which reside in both the gastric mucosa and gastric fluid [21]. In *Helicobacter pylori* (HP)-infected subjects, Proteobacteria is the predominant phylum, comprising 72–99% of all bacteria in the gastric mucosa [12]. In previous work, we showed that HP accounts for 20–98% of all identified gastric mucosal bacteria in infected subjects; in HP-negative individuals, Bacteroidota and Firmicutes are dominant in all gastric sites [22].

Most studies on the upper GI tract microbiota have employed biopsies or brushing samples obtained during upper endoscopy [6,23,24,25]. Non-endoscopic sampling techniques, including the Esophageal String Test [26], inflatable balloons [4], and capsule-sponge devices [27], have also been used in studies of esophageal microbiota. The capsule-sponge device consists of a mesh packed in a capsule that is swallowed and dissolves in the stomach, releasing the sponge, which is withdrawn by pulling the string. Its yield of microbial DNA is >10-fold higher than that of biopsies or brushings, and it provides a more comprehensive view of the esophageal microbiome because samples are collected along the entire length of the esophagus. The capsule-sponge technique has been mainly used for the early diagnosis of Barrett’s metaplasia in the primary care setting [27].

Here, we used 16S rRNA gene amplicon sequencing to compare bacterial diversity and composition in the upper digestive tract between three groups of patients, including functional dyspeptic (FD) patients, patients who underwent curative treatment for head or neck squamous cancer (HNSCC survivors), and patients with histologically proven esophageal squamous intraepithelial neoplasia. The mucosal samples were collected from the oral cavity, esophagus, and gastric corpus using oral swabs, the capsule-sponge device, and gastric endoscopic biopsies, respectively.

## 2. Materials and Methods

### 2.1. Patients

This study enrolled 72 subjects who underwent upper endoscopy for clinical indications. Participants were divided into three patient groups, as follows: (1) 40 FD patients (20 men and 20 women; median ages, 66 and 65 years, respectively; range, 26–76 years), who met the Rome IV criteria defining dyspepsia as any combination of postprandial fullness, early satiety, epigastric pain, and epigastric burning that occur at least 3 days per week over the last 3 months with an onset of at least 6 months in the absence of evidence of organic, structural, systemic or metabolic disease that could explain the symptoms presented [28,29,30]; (2) 21 patients (20 men and 1 woman; median age, 68 years; range 34–80 years) who completed curative treatment for histologically confirmed head and neck squamous cell cancer (HNSCC survivors) of the larynx, oral cavity, oropharynx, or hypopharynx at least 1 year before enrollment (HNSCC survivors); and (3) 11 patients (5 men; median age, 67 years, range 51–80 years, and 6 women; median age, 66 years, range 51–85 years) with histologically proven esophageal squamous intraepithelial neoplasia undergoing qualification for endoscopic treatment. Demographics, clinical data, and anthropometric measures are summarized in Table 1. Subjects were prospectively enrolled over 12 months at a single oncological center, the Maria Sklodowska-Curie National Research Institute of Oncology, Warsaw, Poland. The study was conducted according to the guidelines of the Declaration of Helsinki, and approved by the Bioethics Committee at the Maria Sklodowska-Curie National Research Institute of Oncology (109/PB/2019). Written informed consent for participation in this study was obtained from each participant before the examination.

### 2.2. Procedures

All participants underwent routine upper gastrointestinal endoscopy with gastric biopsy sampling, followed by the collection of capsule-sponge samples (Cytosponge™, Medtronic, Minneapolis, MN, USA) and oral swabs. The gastric biopsy specimens used for histological examination were fixed in buffered 10% formalin, embedded in paraffin, and stored at room temperature. Serial sections were cut and stained with hematoxylin and eosin, and then examined by an experienced pathologist. The capsule-sponge samples were vortexed in BD SurePath liquid at 4 °C, centrifuged to pellet cellular debris (215 g for 5 min), and the residual supernatants were used for DNA extraction. The stomach corpus biopsy specimens, capsule-sponge supernatant samples, and oral swabs, which were used for 16s rRNA sequencing, were flash frozen in liquid nitrogen and stored at –80 °C. All esophageal squamous intraepithelial neoplasia, except one (who underwent definitive Chth/Rth for a T2N0M0 ESCC), ultimately underwent endoscopic removal of neoplastic tissue from the esophagus. Microbial genomic DNA was extracted using a Qiamp DNA Mini Kit (Qiagen, Hilden, Germany) and used for PCR amplification of bacterial 16S hyper-variable regions. Prepared libraries were sequenced using the PGM platform (Thermo Fisher Scientific, Waltham, MA, USA) as described previously [31].

### 2.3. Bioinformatics and Statistical Analysis

Unmapped BAM files were converted to FASTQ using Picard’s SamToFastq v. 1.115 [32]. Additional steps of the analysis were performed using Mothur version 1.47 software [33]. FASTQ files were converted to the FASTA format. For analyses, only sequences 200–300 bp in length, with an average base quality of 20 in a sliding window of 50 bases and a maximum homopolymer length of 10, were kept. Chimeric sequences were identified with the VSEARCH chimera detection algorithm using default parameters [34], with internal sequence collection as the reference database. Chimeric sequences were removed, and the remaining 16S rRNA sequences were classified using the Wang method and the SILVA bacterial 16S rRNA database [35] for reference (release 138), at an 80% bootstrap cut-off. Differential taxa abundance was assessed with a mixed-effects model implementation in LinDA [36]. The non-parametric Shannon diversity index and the Chao richness index were determined with Mothur, and differences in index values were assessed using the Mann–Whitney U-test. Bray–Curtis index analysis and principal coordinate analysis (PCoA) were performed with the vegan package [37]. The ANOSIM test was performed to verify the significance of clustering patterns, differentiating FD patients from neoplasia and HNSCC survivors. For comparison between different sampling sites in FD patients, mixed-effects models on principal component values were used instead. FDR-adjusted [38]. *p*-values ≤ 0.05 were considered statistically significant.

## 3. Results

### 3.1. Patients Overview

The study included three groups of participants who underwent diagnostic gastroscopy: 40 FD patients, 21 HNSCC survivors, and 11 patients with esophageal squamous intraepithelial neoplasia being evaluated for endoscopic resection. Although the FD patients and esophageal neoplasia groups consisted of equal numbers of women and men, HNSCC survivors were predominantly men. Patients’ age and BMI did not differ significantly between the groups (Table 1). No participant had received antibiotic treatment or used PPIs routinely within 1 month prior to sample collection. The quantity and quality of the isolated DNA enabled the construction of libraries from all 72 capsule-sponge samples (all cases), including 67 (39 in FD group, 21 HNSCC survivors, and 7 in esophageal squamous intraepithelial neoplasia groups) gastric biopsy samples, and 54 (40 in FD group, 6 in a group of HNSCC survivors and 8 in esophageal squamous intraepithelial neoplasia groups) oral cavity swabs. Of 67 patients diagnosed by a rapid urease test, 15 (22.4%) were HP positive. After filtering the sequencing data, the median numbers of reads for the gastric corpus mucosa, capsule-sponge samples, and oral cavity swabs were 131,976, 131,482, and 116,837, respectively.

### 3.2. General Bacterial Populations

**Figure 1:**
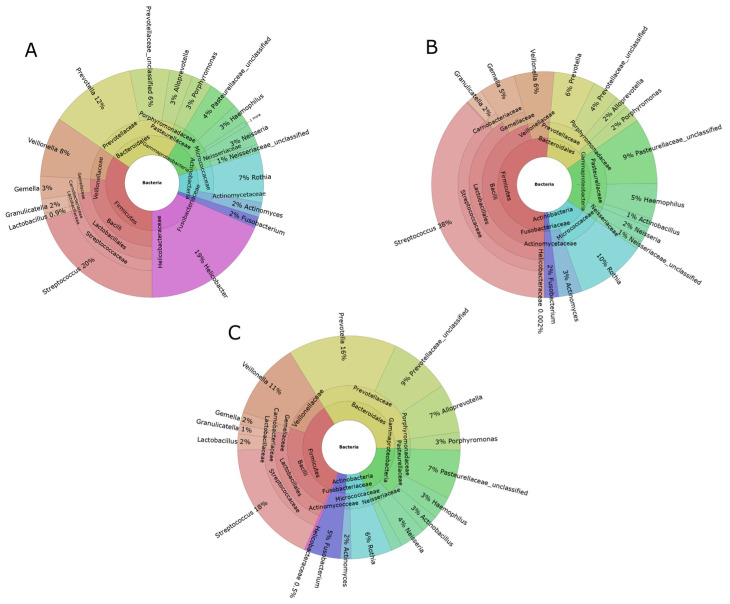
Krona charts of the genera with a mean abundance >1% of the total found in the gastric mucosa (**A**), oral cavity swabs (**B**), and capsule-sponge samples (**C**).

Across all samples, phylum level analysis showed that the bacterial populations could be classified into 39, 33, and 27 phyla in gastric, capsule-sponge, and oral samples, respectively. Of these, Firmicutes, Proteobacteria, Actinobacteriota, Bacteroidota, and Fusobacteriota had a median abundance of >1% in all sites (Figure 1A–C), and Campilobacterota was identified only in gastric and capsule-sponge samples (Figure 1A,C).

### 3.3. Characterization of Microbiota in the FD Group

We first assessed the microbiota of the upper digestive tract in the FD group. The bacterial community structure was evaluated by analyzing α- and β-diversity. The α-diversity was analyzed using the Shannon index, a marker of bacterial richness and evenness, and the Chao index, a marker of richness. The β-diversity was analyzed using PCoA of Bray–Curtis distances. The analyses were performed at the genus level.

**Figure 2:**
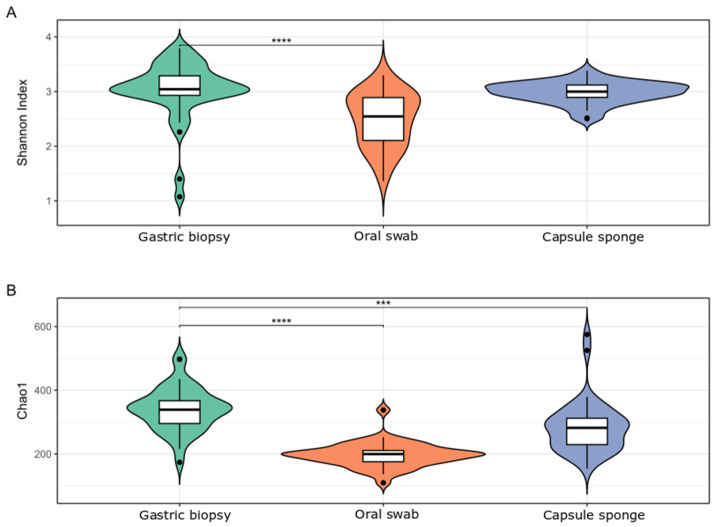
Bacterial α-diversity in gastric, oral, and capsule-sponge samples from the FD group, measured by the Shannon index (**A**) and Chao index (**B**). ***: *p* < 0.001; ****: *p* < 0.0001.

After multiple hypothesis testing corrections, the Shannon index was significantly higher in gastric than in oral samples and did not differ from that in capsule-sponge samples. The Chao index was significantly higher in gastric than in capsule-sponge and oral samples (Figure 2).

**Figure 3:**
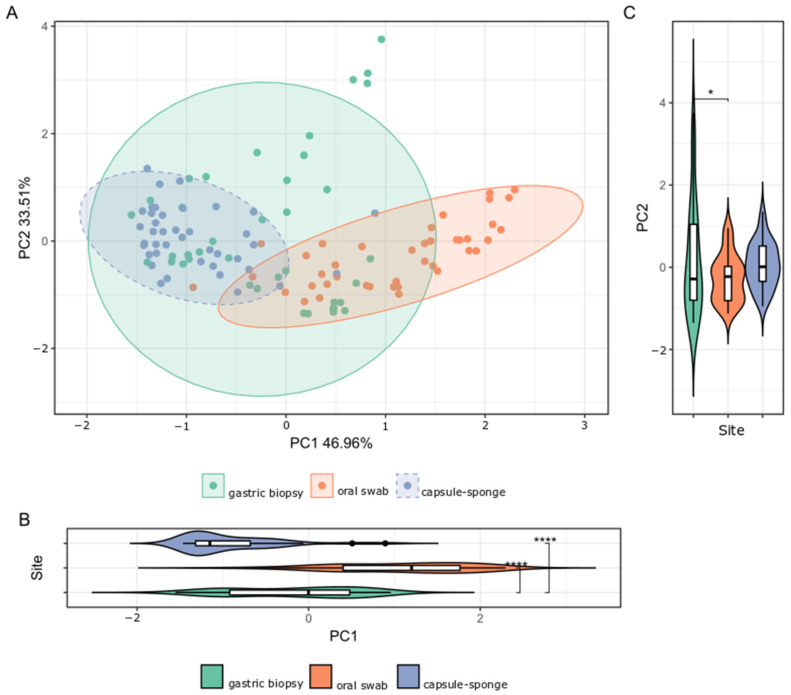
(**A**) β-diversity of the bacterial structure comparing the gastric mucosa, capsule-sponge, and oral microbiome population structure of the FD group, as determined by PCoA analysis based on a Bray–Curtis distance measure. (**B**,**C**) The separation along PC1 and PC2 indicated specific microbial taxa differentiated the gastric mucosa, capsule-sponge, and oral microbiomes. *: *p* < 0.05; ****: *p* < 0.0001.

Despite extensive variability between individual samples from the three sites studied, the microbial compositions of the gastric, capsule-sponge, and oral samples formed three distinct clusters (Figure 3).

The most prevalent genera at the studied sites are presented in Supplementary Table S1. The microbiota of the gastric corpus, as assessed in biopsy samples, was dominated by the Helicobacter genus in 16 (41%) of 39 gastric samples, with the highest frequency in 14 samples and the second highest frequency in 2 samples. By contrast, only 15 (22.4%) of 67 patients diagnosed by rapid urease test were HP positive. Helicobacter was also detected in four (10%) of the capsule-sponge samples, probably as a result of contamination with bacteria from the stomach.

Next, we investigated intergroup differences in the distribution of bacterial genera across sampling methods. There were 242 genera (128 over- and 114 under-represented) that differentiated gastric mucosa from capsule-sponge samples and 228 genera (156 over- and 72 under-represented) that differentiated gastric mucosa from oral cavity samples (adjusted *p*-value (*p* adj) < 0.05; Supplementary Table S1). There were 45 genera unique to oral swabs and 59 unique to capsule-sponge samples. Up to 183 genera were common in the comparison of swabs and capsule-sponge vs. gastric biopsies (*p* adj < 0.05; Supplementary Table S2).

All the differential bacteria were over-represented in capsule-sponge samples. Eighty-eight taxa differentiated capsule-sponge from gastric biopsies, and most of them (76) were over-represented in biopsies. Finally, 206 taxa were over-represented in biopsies compared with oral populations. Taken together, these findings indicate that bacterial populations differ among the studied sites at the genus level.

**Figure 4:**
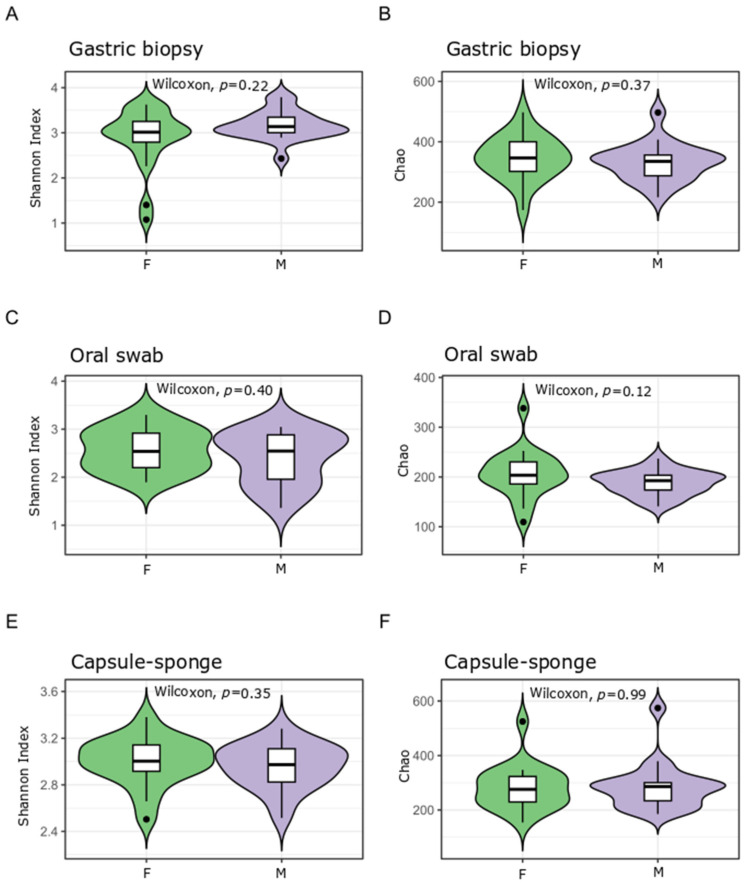
Bacterial α-diversity in the capsule-sponge, gastric corpus biopsy, and oral swab samples collected from men (M) and women (F) in the FD group and measured by the Shannon index (**A**,**C**,**E**) and Chao index (**B**,**D**,**F**).

To determine whether the bacterial composition and taxonomy of the gastric, capsule-sponge, and oral samples may be sex-dependent, we repeated the analyses in the sex-disaggregated FD group. Both Shannon and Chao indexes did not differ between women and men (Figure 4).

**Figure 5:**
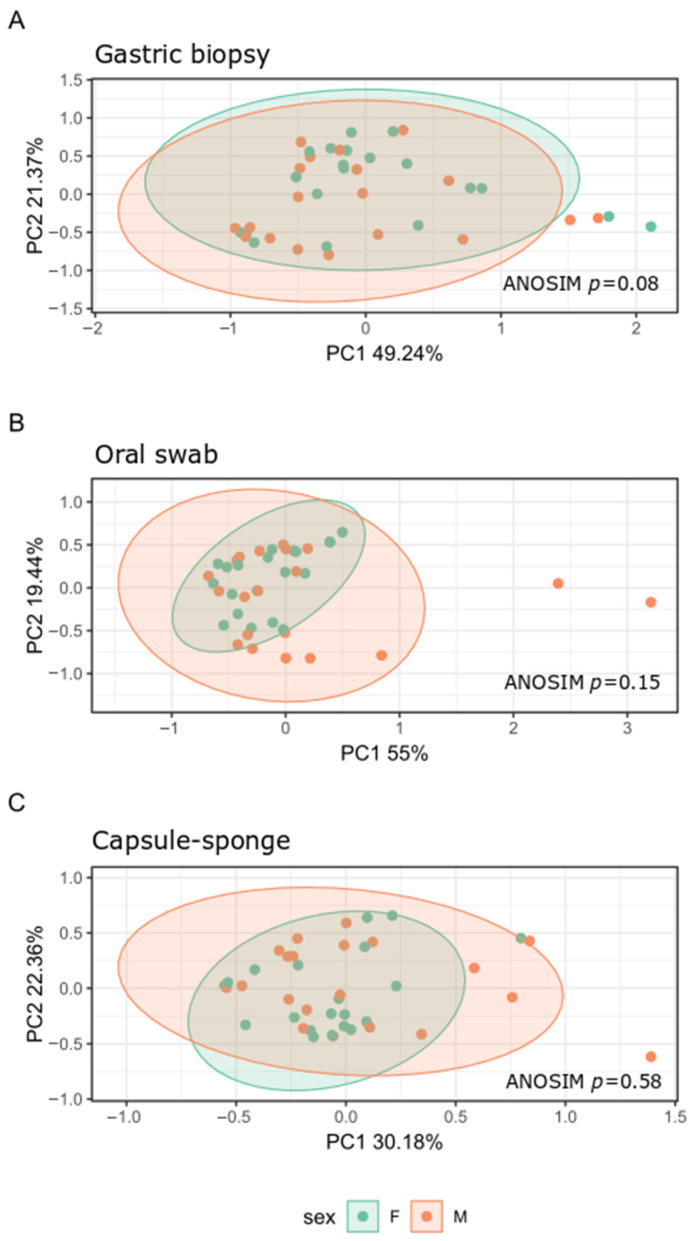
β-diversity of bacterial structure evaluating similarities between the gastric mucosa (**A**), oral microbiota (**B**) and capsule-sponge (**C**) structure of men (M) and women (F) in the FD group, as determined by PCoA analysis based on a Bray–Curtis distance measure.

Analysis of the β-diversity of bacterial communities showed separate clusters with different microbial compositions between men and women only in the gastric biopsy samples (Figure 5) (*p* adj = 0.08). The relative abundance of only one genus, *Veillonellaceae unclassified*, which was under-represented in men, distinguished men from women (log2 Fold Change = −2.08; *p* adj = 0.0399) in the oral site. Thus, the microbiota structure of the upper GI tract did not differ between men and women.

To summarize, in the FD group, the microbiota diversity was comparable between gastric and capsule-sponge samples, whereas oral cavity samples showed lower diversity. We observed differences in β-diversity between samples, demonstrating that the devices used were capable of sampling a variety of microbiomes. These results were irrespective of patients’ sex.

### 3.4. Microbiota Analysis of the HNSCC Survivors and Esophageal Squamous Intraepithelial Neoplasia Patients

**Figure 6:**
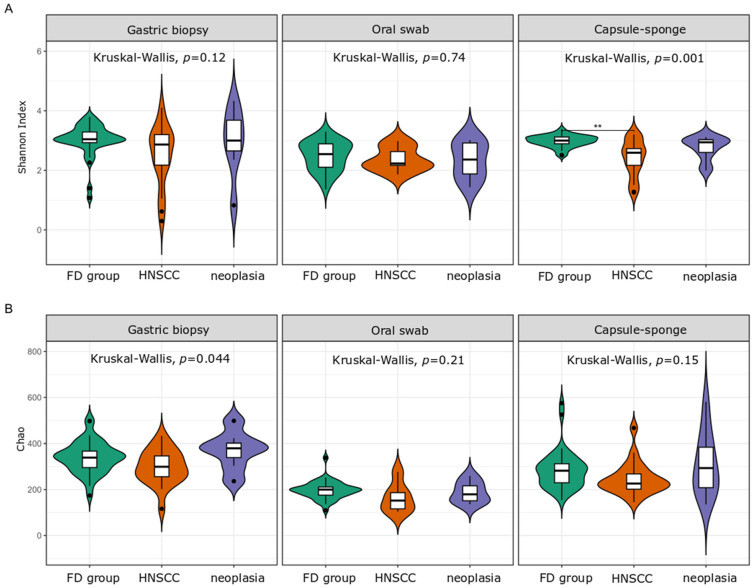
Bacterial α-diversity in the gastric corpus mucosa, oral swab samples, and the capsule-sponge as measured by the Shannon index (**A**) and Chao index (**B**), comparing the bacterial community structure between the studied groups. FD group, functional dyspepsia group; HNSCC, head and neck squamous cell cancer survivors; neoplasia, esophageal squamous intraepithelial neoplasia. **: *p* < 0.01.

**Figure 7:**
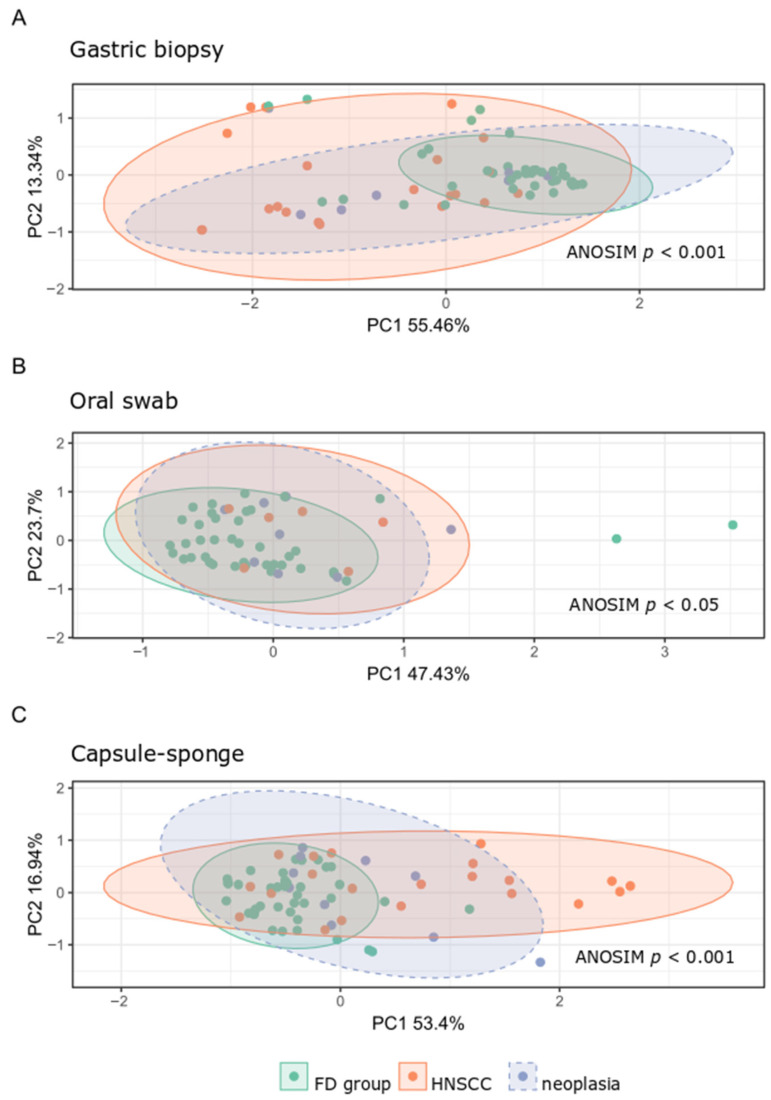
β-diversity of bacterial structure in the studied groups from gastric biopsy (**A**), oral samples (**B**) and capsule-sponge (**C**). FD group, functional dyspepsia group; HNSCC, head and neck squamous cell cancer survivors; neoplasia, esophageal squamous intraepithelial neoplasia.

Next, we performed a comparative analysis of the collected samples according to sampling method and patient status. We observed only minor changes in the α-diversity. The Shannon index of the capsule-sponge samples was significantly higher in the FD group than in patients after curative treatment for HNSCC, and the Chao index within gastric corpus samples differentiated patients after HNSCC treatment from the FD group (Figure 6). By contrast, β-diversity, which evaluated similarities between the microbiome population structure of the FD group, HNSCC survivors, and esophageal squamous intraepithelial neoplasia patients, showed distinct clusters formed at each location (Figure 7).

The relative abundance of 205, 116, and 9 bacterial genera in gastric, capsule-sponge, and oral samples, respectively, was significantly different between the FD group and HNSCC survivors. In gastric biopsy samples, 33 genera were different between the FD group and patients with esophageal squamous intraepithelial neoplasia (*p* adj < 0.05; Supplementary Table S3). Only three genera in the capsule-sponge samples (*Lactobacillus*, *Salmonella*, *and Thermus*) and three genera in the swab samples (*Gammaproteobacteria unclassified*, *Haemophilus*, and *Bifidobacterium*) differentiated esophageal squamous intraepithelial neoplasia patients from the FD group, although the difference was not statistically significant after correcting for multiple testing (*p* adj = 0.067). The frequency of *Helicobacter* genus infection was comparable among groups; the *Helicobacter* genus was predominant in eight (21.5%), seven (33.3%), and one (14.3%) sample in the FD patients, HNSCC survivors, and esophageal squamous intraepithelial neoplasia patient groups, respectively.

Among bacteria differentiating HNSCC survivors from the FD group in gastric corpus samples, 155 genera (out of 205) were under-represented in HNSCC survivors, including *Actinobacillus*, *Treponema*, *Lautropia*, *Tannerella*, *Bilophila*, *Alistipes*, *Aggregatibacter*, *Bibersteinia*, *Selenomonas*, and *Leptotrichia*. The remaining 50 were over-represented in HNSCC patients, including *Rheinheimera*, *Renibacterium*, *Pedobacter*, *Ralstonia*, *Gardnerella*, *Rhodoluna*, *Paucibacter Pelomonas*, and *Bacillus*.

**Figure 8:**
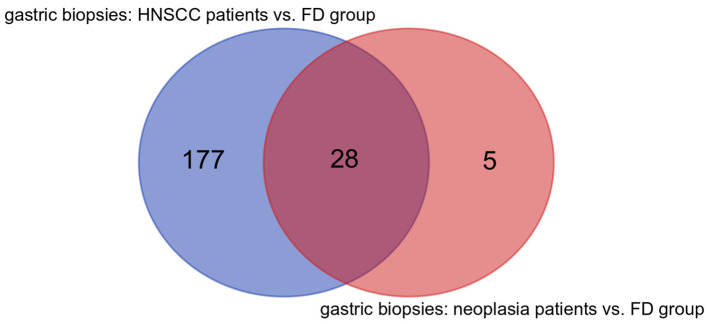
Common and unique genera characteristic to gastric biopsies in head and neck squamous cell cancer (HNSCC) survivors and esophageal squamous intraepithelial neoplasia vs. FD group comparisons.

Of 33 genera, 13 were over-represented (including *Mycobacterium, Polynucleobacter, Caulobacter, Finegoldia*, and *Hyphomicrobium*) and 20 were under-represented (including *Actinobacillus, Treponema, Butyrivibrio, Filifactor*, *Mycoplasma*, and *Eikenella*) in esophageal squamous intraepithelial neoplasia patients compared with FD group (Supplementary Table S3). Furthermore, 28 differential genera were identified in both groups of patients vs. FD group comparisons. Five bacterial genera were unique to the comparison of esophageal squamous intraepithelial neoplasia vs. FD patients, and all of these were over-represented (*Finegoldia, Rhodocyclaceae_C39, Mycobacterium, Modestobacter*, and *Hyphomicrobium*) in esophageal squamous intraepithelial neoplasia patients. By contrast, as many as 177 taxa were unique to the HNSCC survivors vs. FD patients comparison (Figure 8).

In the capsule-sponge samples, 20 differential bacteria were over-represented (including *Lactobacillus*, *Ralstonia*, *Arcanobacterium*, *Rhodoferax*, *Staphylococcus*, *Streptococcus*, and *Rothia*) and 96 were under-represented in HNSCC survivors compared with the FD group (including *Lautropia*, *Bulleidia*, *Catonella*, *Peptococcus*, *Cardiobacterium*, *Prevotella*, *Neisseria*, *Leptotricha*, *Megasphaera*, *Fusobacterium*, *Bibersteinia*, *Aggregatibacter*, *Tannerella*, *Treponema*, *Actinobacillus*, *Tuzzerella*, *Peptococcus*, *Campylobacter*, and *Dialister*). By contrast, all oral swab bacteria differentiating HNSCC survivors from the FD group, including *Lautropia*, *Corynebacterium*, and *Cardiobacterium*, were under-represented in HNSCC survivors.

**Figure 9:**
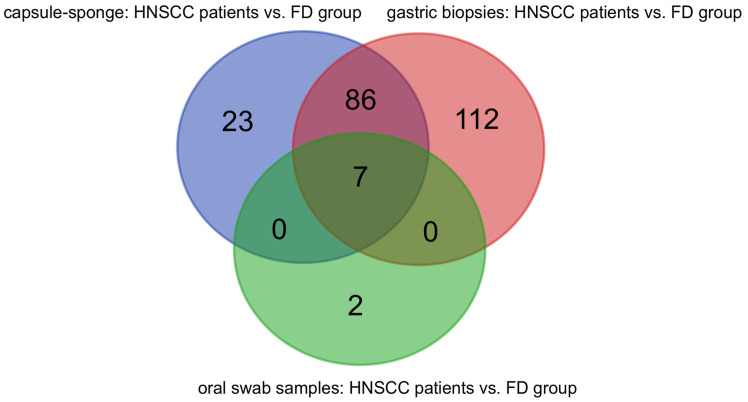
Common and unique genera in gastric biopsy, capsule-sponge, and oral swab samples in head and neck squamous cell cancer (HNSCC) survivors vs. FD group comparisons.

**Figure 10:**
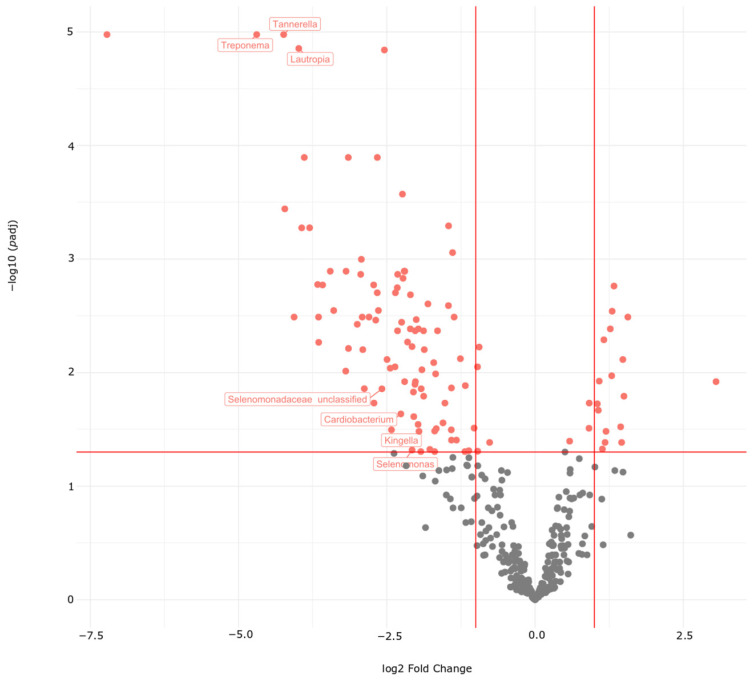
Volcano plot of bacterial taxa present in capsule-sponge samples. Differential taxa between the FD group and HNSCC survivors are marked in red. Labeled taxa are differential in gastric biopsy and oral swabs as well. Lines—thresholds for *p* < 0.05 and fold change > 2.

Next, we searched for unique and common bacteria in the comparisons of HNSCC survivors vs. FD group in samples from gastric biopsies, capsule-sponge device, and oral swabs (Supplementary Table S4). Seven bacterial genera (*Selenomonas*, *Treponema*, *Selenomonadaceae unclassified*, *Cardiobacterium*, *Lautropia*, *Tannerella*, and *Kingella*) were common to the three collection sites (Figure 9 and Figure 10). We identified 86 genera common to capsule-sponge and gastric biopsy samples, whereas 23, 112, and 2 genera were unique to capsule-sponge, gastric biopsy, and oral swab samples, respectively (Figure 9).

## 4. Discussion

The incidence of ESCC shows wide geographical variation, with rates of up to 21.62 cases per 100,000 population in certain high-incidence areas of China [39]. In Western countries, the incidence of ESCC is low, except in high-risk individuals, which include patients who underwent endoscopic resection of ESCC and those following curative treatment for HNSCC [23]. Nutrition plays an important role in the health and recovery of cancer patients [40]. Malnutrition can affect treatment responses, reduce immune function, and delay recovery [41]. In HNSCC patients, similar risk factors, such as excessive alcohol intake and smoking [42], not only contribute to developed synchronous and metachronous ESCC but also increase the risk of malnutrition [43]. Addressing these issues through proper nutritional support may help improve treatment outcomes and overall patient well-being. Magnano et al. [44] showed that patients with head and neck cancer are particularly vulnerable to malnutrition. The Nutritional Risk Index identified 96 and 42 out of 144 patients as severely and moderately malnourished, respectively, regardless of their weight status. In contrast, the use of BMI underestimated malnutrition, especially in overweight and obese individuals [44]. The composition of the microbiota, particularly in the upper digestive tract, can be profoundly affected by dietary habits, impacting both disease progression and treatment outcomes [45]. A well-balanced diet not only supports the immune system and overall health but also helps maintain a diverse and stable microbiome, which can influence the body’s ability to respond to cancer therapies and recover post-treatment [46]. Additionally, specific dietary interventions can modulate the microbiome in ways that reduce inflammation, enhance the efficacy of treatments, and lower the risk of recurrence [47].

In this study, we compared the microbiota composition of the upper digestive tract between individuals with FD and patients with histologically proven esophageal squamous neoplasia and those who completed curative treatment for HNSCC at least one year before the study. 16S rRNA gene amplicon sequencing was performed using DNA samples isolated from an endoscopic biopsy of the gastric corpus mucosa, oral swabs, and capsule-sponge samples, which yielded the highest DNA quantities.

The similarities and differences in bacterial community structure and taxonomy between different sites of the upper digestive tract were analyzed using sequencing data collected from the FD group. Bacterial richness and evenness, evaluated by the Shannon index, were significantly higher in gastric samples than in oral samples, but did not differ between gastric samples and capsule-sponge samples. The Chao index, a marker of bacterial richness, was significantly higher in gastric samples than in capsule-sponge and oral samples. Consistently, PCoA analysis of the β-diversity of bacterial communities showed three separate clusters that significantly distinguished the microbial composition of gastric samples from that of capsule-sponge and oral swab samples. Taxonomic analyses identified 242 and 228 bacterial genera whose relative abundance differentiated gastric samples from capsule-sponge and oral samples, respectively. These results indicate that the bacterial communities of the upper digestive tract are clustered, at least partially, according to the anatomic site, and the oral cavity, esophagus, and stomach are likely hosts to distinct microbial communities. In the presence/absence testing, the biggest differences were observed between oral and gastric samples.

Sex-related differences were minimal in our study and confined only to oral cavity samples. This is in contrast with previous studies reporting significant sex-related differences in bacterial diversity and composition in both oral [48,49] and gastric microbiota [50]. ESCC shows a strong male predominance [51], and the oral microbiome composition prospectively predicts the risk for ESCC [52]. However, a causal relationship remains to be determined, and the present study did not identify sex-related bacteria, which is most likely due to the limited sample size.

A low microbial richness in the upper digestive tract is independently associated with esophageal squamous dysplasia [4]; however, the present data did not confirm these findings. Instead, we showed that the Shannon index was lower in HNSCC survivors than in the FD group. In the two other sites, there were no differences in the microbial α-diversity between the FD patients and two other groups. By contrast, the β-diversity of the bacterial population structure differed significantly between FD patients and the other groups at each site studied. In the gastric, capsule-sponge, and oral samples, 205, 116, and 9 bacterial genera differentiated FD patients from HNSCC survivors, whereas only 33 genera differentiated FD patients from esophageal squamous intraepithelial neoplasia patients in capsule-sponge samples. Thus, although limited differences in microbial community membership or diversity were found between FD patients individuals and patients with esophageal squamous intraepithelial neoplasia, substantial differences in gastric and esophageal microbiota samples were observed between FD patients and patients who were previously treated for HNSCC.

As reported previously, the oral microbiome is dominated by six phyla, *Firmicutes*, *Bacteroidetes*, *Proteobacteria*, *Actinobacteria*, *Spirochaetes*, and *Fusobacteria*, accounting for 96% of the taxa showing relatively stable abundance at the genus level [9,53]. Major phyla detected in the esophagus include *Firmicutes*, *Bacteroidetes*, *Actinobacteria*, *Proteobacteria*, *Fusobacteria*, and *TM*7 [54,55,56], and differences in the relative abundance of taxa are found between the oral cavity and normal esophagus, dominated by *Streptococcus*, *Prevotella*, and *Veillonella* [9]. Neither microbiota composition nor diversity differentiates the upper, middle, and lower segments of the normal esophagus [54,57]. *Proteobacteria* is the predominant phylum in HP-infected subjects, accounting for 72–99% of all bacteria in the gastric mucosa. Regardless of the HP status, other phyla, including *Actinobacteria*, *Bacteroidetes*, *Firmicutes*, and *Fusobacteria*, are also detected consistently [12]. The predominant bacteria in the gastric mucosa are *Helicobacter*, *Streptococcus*, *Rothia*, *Lactobacillus*, *Veillonella*, *Prevotella*, *Neisseria*, *Hemophilus*, and others, depending on the HP status [58]. However, because the microbiome structure varies significantly among body sites, defining a “normal” microbiome is challenging, not just in the stomach, esophagus, or mouth [24]. In addition, the bacterial composition of the upper digestive tract depends on various factors, including age, diet, alcohol and smoking, acid reflux, treatment with proton pump inhibitors (PPIs), and chronic gastritis [25].

Microbial imbalance or dysbiosis is associated with different pathologies, including inflammation and cancer. Gastric microbiota dysbiosis may induce and maintain carcinogenic pathways by promoting inflammation, increased cell proliferation, dysregulation of stem cell physiology, and production of cancerogenic metabolites, and *H. pylori* is considered a class I carcinogen [20]. In this study, *H. pylori* was dominant in 21.5%, 33.3%, and 14.3% of gastric samples from the FD patients, HNSCC survivors, and esophageal squamous intraepithelial neoplasia groups, respectively.

A few studies examined the esophageal microbiome in ESCC. Shao et al. [18] analyzed microbial communities in paired tumor and non-tumor samples from patients with ESCC and gastric cardia adenocarcinoma recruited from geographic regions of China with a high incidence of ESCC. They found similar taxa present in ESCC tumor and non-tumor samples, with a decreased relative abundance of *Streptococcus* and an increased level of *Fusobacterium* in ESCC samples. Yu et al. [4] reported a lower esophageal microbial richness associated with dysplasia, whereas Li et al. [59] showed that ESCC patients exhibit lower microbial richness and evenness than controls, and an increased abundance of *Streptococcus*, *Lactobacillus*, *Prevotella*, and *Fusobacterium*; the key taxa in the esophageal microbiome of ESCC patients, as identified by unweighted UniFrac, were *Clostridiales*, *Pseudomonas*, and *Selenomonadales*. Nasrollahzadeh et al. [60] compared the gastric corpus microbiome between early ESCC (stage I–II) and esophageal squamous dysplasia Iranian patients and controls and found that α-diversity did not distinguish cases from controls; however, the abundance of *Clostridiales* and *Erysipelotrichales* was significantly higher in early ESCC patients than in healthy controls. In esophageal adenocarcinoma tissues, decreased microbial diversity with a decreased abundance of Gram-negative (*Veillonella*, *Megasphaera*, and *Campylobacter*) and Gram-positive taxa (*Granulicatella*, *Atopobium*, *Actinomyces*, and *Solobacterium*) and a high abundance of *Lactobacillus* spp. and *Streptococcus* spp. were reported [20], whereas another study showed only a modest reduction in diversity in BE and no genera differentiating controls from BE or BE from esophageal adenocarcinoma [27].

In this study, we found a decreased abundance of *Lactobacillus*, *Veillonella*, *Megasphaera*, and *Campylobacter* in gastric biopsy and capsule-sponge samples in HNSCC survivors compared with the FD group. *Lactobacillus* bacteria were also more abundant in the group of patients with esophageal squamous intraepithelial neoplasia, although the result did not reach statistical significance. The genus *Streptococcus*, on the other hand, was more abundant in capsule-sponge samples from HNSCC survivors. The present findings from capsule-sponge samples in HNSCC survivors were similar to those of Li et al., who reported that in both saliva and cell brush specimens, the *Granulicatella*, *Rothia*, *Streptococcus*, *Gemella*, *Leptotrichia*, and *Schaalia* genera were common biomarkers in patients with esophageal low-grade dysplasia, whereas *Lactobacillus* was a common biomarker in patients with high-grade dysplasia [59]. In addition, the abundance of *Streptococcus* and *Prevotella* is associated with advanced esophageal cancer [61]. We found increased *Streptococcus* and decreased *Prevotella* abundance in capsule-sponge samples from the HNSCC group. Li et al. showed that the abundance of *Prevotella* was significantly lower in ESCC patients [59]. It remains unknown whether esophageal dysbiosis is a cause or an effect of esophageal oncogenesis. Yang et al. observed a significant over-representation of *Fusobacterium* in ESCC tissues [62], whereas in the present study, the relative abundance of *Fusobacterium* was decreased in both gastric biopsies and capsule-sponge samples in HNSCC survivors compared with FD group.

Previous studies showed that *Porphyromonas gingivalis* is present in the cancerous and surrounding esophageal mucosa of patients with ESCC but absent in the healthy mucosa of control subjects [63]. Moreover, the presence of *Porphyromonas gingivalis* is closely associated with the severity of ESCC. These findings suggest that *Porphyromonas gingivalis* could potentially serve as a valuable biomarker for predicting and monitoring ESCC. In this study, we showed that *Porphyromonas* abundance was lower in HNSCC survivors than F in both gastric biopsies and capsule-sponge samples, which could be related to previous oncological treatment. Chen et al. reported changes in the bacterial microbiota present in saliva that were likely associated with ESCC development [64]. The present findings are consistent with those of Chen et al. In HNSCC individuals, we observed a lower abundance of *Lautropia*, *Corynebacterium*, and *Cardiobacterium* genera in oral swab samples. Additionally, we showed a noticeable decrease in the abundance of these genera in both gastric biopsies and capsule-sponge samples in HNSCC survivors compared with the FD group.

The results of this study showed slight differences in microbial community composition or diversity between FD patients and both other groups in oral cavity samples, whereas substantial differences were observed in gastric samples, particularly between FD and HNSCC survivor groups. Compared with FD patients, substantial differences were also noted in capsule-sponge samples from HNSCC survivors, but not in those from patients with esophageal squamous intraepithelial neoplasia. However, the capsule-sponge device samples the entire length of the esophagus, as well as the proximal stomach and oral cavity [27]. This may cause the microbiome of neoplastic tissue to be diluted by the microbiome of the normal tissues of the upper digestive tract. The capsule-sponge device yielded a 10-fold greater abundance of microbial DNA than biopsies/brushings [27].

One limiting factor of this microbiota study was the lack of information on certain potential gastric and esophageal dysbiosis confounders, including dietary and lifestyle habits, and particularly the use of acid-suppressant drugs. PPIs may alter the upper digestive tract microbiome both by increasing the pH of gastric secretions and by directly targeting the bacterial proton pumps of certain bacteria that contain P-type ATPase enzymes [6]. Other limitations include the relatively small sample size of the esophageal squamous intraepithelial neoplasia, the lack of uniformity in the treatment regiments and duration, which included both chemo- and radiotherapy, among the HNSCC survivors, and the lack of samples from healthy individuals which meant that we had to replace samples from healthy volunteers with samples from a heterogeneous group of patients with dyspeptic symptoms.

## 5. Conclusions

Nevertheless, the results of this study suggest that patients who have undergone curative treatment for HNSCC exhibit significant dysbiosis of the upper digestive tract. Whether gastric and esophageal dysbiosis is associated with or is a consequence of HNSCC treatment remains unclear. To resolve this issue, further prospective studies are needed, for which the capsule-sponge device may be a useful tool. Because the capsule-sponge device is a convenient non-endoscopic sampling procedure to collect bacteria from the gastric corpus mucosa and the entire upper digestive tract, it may also be useful for studying the action of external influences, such as diet and lifestyle, on tumor development and progression.

## Figures and Tables

**Table 1:** Demographics, clinical data, and anthropometric measures.

Characteristics	FD Patients	HNSCC Survivors	Esophageal Squamous Intraepithelial Neoplasia
Number of cases	40	21	11
Age: mean (±SD) years	59.2 (±13.9)	66.6 (±10.9)	66.0 (±10.3)
Sex: male; n (%)	20 (50%)	20 (95.2%)	5 (45.5%)
BMI: median cm/m^2^ (IQR)	26.2 (24.6–28.3)	26.5 (25.7–27.9)	23.4 (21.5–26.4)
Smoking: active and former; n (%)	17 (42.5%)	18 (85.7%)	7 (63.6%)
Tumor location: n (%)	N/A	Primary tumor site Oral cavity: 3 (14.3%) Oropharynx: 8 (38.1%) Larynx: 10 (47.6%)	Esophagus Proximal: 3 (27.3%) Middle: 5 (45.4%) Distal: 3 (27.3%)
Histopathology: n (%)	N/A	Squamous cell carcinoma: 21 (100%)	HG-IEN: 3 (27.3%) pT1a ESCC: 2 (18.2%) pT1b ESCC: 5 (45.5%) T2 ESCC: 1 (9.0%)

BMI, body mass index; ESCC, esophageal squamous cell carcinoma; FD patients, functional dyspeptic patients; HNSCC, head and neck squamous cell cancer; HG-IEN, high grade intraepithelial neoplasia; IQR, interquartile range; N/A, not applicable; SD, standard deviation.

## Data Availability

The data presented in this study are openly available in BioProject https://www.ncbi.nlm.nih.gov at PRJNA1048486.

## Supplementary Materials

The following supporting information can be downloaded at https://www.mdpi.com/article/10.3390/cancers16203528/s1. Table S1: Intergroup differences in the distribution of bacterial genera across sampling methods: capsule-sponge and oral samples vs. gastric biopsies. Table S2: Unique and common bacteria in the distribution of bacterial genera across sampling methods. Table S3: Bacterial genera differentiating HNSCC patients from the FD group in gastric biopsies, capsule-sponge, and oral samples. Table S4: Unique and common bacteria in the comparisons of HNSCC vs. FD group in samples from gastric biopsies, capsule-sponge, and oral samples.

## References

[1] She J.-J., Liu W.-X., Ding X.-M., Guo G., Han J., Shi F.-Y., Lau H.C.-H., Ding C.-G., Xue W.-J., Shi W. (2024). Defining the Biogeographical Map and Potential Bacterial Translocation of Microbiome in Human “Surface Organs”. Nat. Commun..

[2] Nardone G., Compare D., Rocco A. (2017). A Microbiota-Centric View of Diseases of the Upper Gastrointestinal Tract. Lancet Gastroenterol. Hepatol..

[3] Wang G. (2024). Advancement of the Relationship between Esophageal Microorganisms and Esophageal Diseases. Gastroenterol. Endosc..

[4] Yu G., Gail M.H., Shi J., Klepac-Ceraj V., Paster B.J., Dye B.A., Wang G.-Q., Wei W.-Q., Fan J.-H., Qiao Y.-L. (2014). Association between Upper Digestive Tract Microbiota and Cancer-Predisposing States in the Esophagus and Stomach. Cancer Epidemiol. Biomark. Prev..

[5] Liu N., Ando T., Ishiguro K., Maeda O., Watanabe O., Funasaka K., Nakamura M., Miyahara R., Ohmiya N., Goto H. (2013). Characterization of Bacterial Biota in the Distal Esophagus of Japanese Patients with Reflux Esophagitis and Barrett’s Esophagus. BMC Infect. Dis..

[6] May M., Abrams J.A. (2018). Emerging Insights into the Esophageal Microbiome. Curr. Treat. Options Gastroenterol..

[7] Ridaura V.K., Faith J.J., Rey F.E., Cheng J., Duncan A.E., Kau A.L., Griffin N.W., Lombard V., Henrissat B., Bain J.R. (2013). Gut Microbiota from Twins Discordant for Obesity Modulate Metabolism in Mice. Science.

[8] Segata N., Haake S.K., Mannon P., Lemon K.P., Waldron L., Gevers D., Huttenhower C., Izard J. (2012). Composition of the Adult Digestive Tract Bacterial Microbiome Based on Seven Mouth Surfaces, Tonsils, Throat and Stool Samples. Genome Biol..

[9] Dewhirst F.E., Chen T., Izard J., Paster B.J., Tanner A.C.R., Yu W.-H., Lakshmanan A., Wade W.G. (2010). The Human Oral Microbiome. J. Bacteriol..

[10] Maukonen J., Mättö J., Suihko M.-L., Saarela M. (2008). Intra-Individual Diversity and Similarity of Salivary and Faecal Microbiota. J. Med. Microbiol..

[11] Andersson A.F., Lindberg M., Jakobsson H., Bäckhed F., Nyrén P., Engstrand L. (2008). Comparative Analysis of Human Gut Microbiota by Barcoded Pyrosequencing. PLoS ONE.

[12] Bik E.M., Eckburg P.B., Gill S.R., Nelson K.E., Purdom E.A., Francois F., Perez-Perez G., Blaser M.J., Relman D.A. (2006). Molecular Analysis of the Bacterial Microbiota in the Human Stomach. Proc. Natl. Acad. Sci. USA.

[13] Gall A., Fero J., McCoy C., Claywell B.C., Sanchez C.A., Blount P.L., Li X., Vaughan T.L., Matsen F.A., Reid B.J. (2015). Bacterial Composition of the Human Upper Gastrointestinal Tract Microbiome Is Dynamic and Associated with Genomic Instability in a Barrett’s Esophagus Cohort. PLoS ONE.

[14] Zou Q., Feng L., Cai X., Qian Y., Xu L. (2023). Esophageal Microflora in Esophageal Diseases. Front. Cell. Infect. Microbiol..

[15] Yang L., Lu X., Nossa C.W., Francois F., Peek R.M., Pei Z. (2009). Inflammation and Intestinal Metaplasia of the Distal Esophagus Are Associated with Alterations in the Microbiome. Gastroenterology.

[16] Blackett K.L., Siddhi S.S., Cleary S., Steed H., Miller M.H., Macfarlane S., Macfarlane G.T., Dillon J.F. (2013). Oesophageal Bacterial Biofilm Changes in Gastro-Oesophageal Reflux Disease, Barrett’s and Oesophageal Carcinoma: Association or Causality?. Aliment. Pharmacol. Ther..

[17] Hayashi H., Sakamoto M., Benno Y. (2002). Phylogenetic Analysis of the Human Gut Microbiota Using 16S rDNA Clone Libraries and Strictly Anaerobic Culture-Based Methods. Microbiol. Immunol..

[18] Shao D., Vogtmann E., Liu A., Qin J., Chen W., Abnet C.C., Wei W. (2019). Microbial Characterization of Esophageal Squamous Cell Carcinoma and Gastric Cardia Adenocarcinoma from a High-Risk Region of China. Cancer.

[19] Amir I., Konikoff F.M., Oppenheim M., Gophna U., Half E.E. (2014). Gastric Microbiota Is Altered in Oesophagitis and Barrett’s Oesophagus and Further Modified by Proton Pump Inhibitors. Environ. Microbiol..

[20] Hunt R.H., Yaghoobi M. (2017). The Esophageal and Gastric Microbiome in Health and Disease. Gastroenterol. Clin. N. Am..

[21] Minalyan A., Gabrielyan L., Scott D., Jacobs J., Pisegna J.R. (2017). The Gastric and Intestinal Microbiome: Role of Proton Pump Inhibitors. Curr. Gastroenterol. Rep..

[22] Ostrowski J., Kulecka M., Zawada I., Żeber-Lubecka N., Paziewska A., Graca-Pakulska K., Dąbkowski K., Skubisz K., Cybula P., Ambrożkiewicz F. (2021). The Gastric Microbiota in Patients with Crohn’s Disease; a Preliminary Study. Sci. Rep..

[23] Januszewicz W., Fitzgerald R.C. (2019). Early Detection and Therapeutics. Mol. Oncol..

[24] Yano Y., Etemadi A., Abnet C.C. (2021). Microbiome and Cancers of the Esophagus: A Review. Microorganisms.

[25] Park C.H., Lee S.K. (2020). Exploring Esophageal Microbiomes in Esophageal Diseases: A Systematic Review. J. Neurogastroenterol. Motil..

[26] Fillon S.A., Harris J.K., Wagner B.D., Kelly C.J., Stevens M.J., Moore W., Fang R., Schroeder S., Masterson J.C., Robertson C.E. (2012). Novel Device to Sample the Esophageal Microbiome—The Esophageal String Test. PLoS ONE.

[27] Elliott D.R.F., Walker A.W., O’Donovan M., Parkhill J., Fitzgerald R.C. (2017). A Non-Endoscopic Device to Sample the Oesophageal Microbiota: A Case-Control Study. Lancet Gastroenterol. Hepatol..

[28] Francis P., Zavala S.R. (2024). Functional Dyspepsia. StatPearls.

[29] Stanghellini V. (2017). Functional Dyspepsia and Irritable Bowel Syndrome: Beyond Rome IV. Dig. Dis. Basel Switz..

[30] Stanghellini V., Chan F.K.L., Hasler W.L., Malagelada J.R., Suzuki H., Tack J., Talley N.J. (2016). Gastroduodenal Disorders. Gastroenterology.

[31] Zeber-Lubecka N., Kulecka M., Lindner B., Krynicki R., Paziewska A., Nowakowski A., Bidzinski M., Ostrowski J. (2022). Increased Diversity of a Cervical Microbiome Associates with Cervical Cancer. Front. Oncol..

[32] Picard Tools—By Broad Institute. https://broadinstitute.github.io/picard/.

[33] Schloss P.D., Westcott S.L., Ryabin T., Hall J.R., Hartmann M., Hollister E.B., Lesniewski R.A., Oakley B.B., Parks D.H., Robinson C.J. (2009). Introducing Mothur: Open-Source, Platform-Independent, Community-Supported Software for Describing and Comparing Microbial Communities. Appl. Environ. Microbiol..

[34] Rognes T., Flouri T., Nichols B., Quince C., Mahé F. (2016). VSEARCH: A Versatile Open Source Tool for Metagenomics. PeerJ.

[35] Quast C., Pruesse E., Yilmaz P., Gerken J., Schweer T., Yarza P., Peplies J., Glöckner F.O. (2013). The SILVA Ribosomal RNA Gene Database Project: Improved Data Processing and Web-Based Tools. Nucleic Acids Res..

[36] Zhou H., He K., Chen J., Zhang X. (2022). LinDA: Linear Models for Differential Abundance Analysis of Microbiome Compositional Data. Genome Biol..

[37] Dixon P. (2003). VEGAN, a Package of R Functions for Community Ecology. J. Veg. Sci..

[38] Benjamini Y., Hochberg Y. (1995). Controlling the False Discovery Rate: A Practical and Powerful Approach to Multiple Testing. J. R. Stat. Soc. Ser. B Methodol..

[39] Zeng H., Zheng R., Zhang S., Zuo T., Xia C., Zou X., Chen W. (2016). Esophageal Cancer Statistics in China, 2011: Estimates Based on 177 Cancer Registries. Thorac. Cancer.

[40] Vitaloni M., Caccialanza R., Ravasco P., Carrato A., Kapala A., de van der Schueren M., Constantinides D., Backman E., Chuter D., Santangelo C. (2022). The Impact of Nutrition on the Lives of Patients with Digestive Cancers: A Position Paper. Support. Care Cancer.

[41] Munteanu C., Schwartz B. (2022). The Relationship between Nutrition and the Immune System. Front. Nutr..

[42] Chaber-Ciopinska A., Kiprian D., Wieszczy P., Bielasik A., Bugajski M., Januszewicz W., Jarzabski A., Niemiec M., Mroz A., Kawecki A. (2023). Narrow Band Imaging versus Lugol Chromoendoscopy in Screening for Esophageal Squamous Neoplasia: A Randomized Trial. Pol. Arch. Intern. Med..

[43] Susetyowati S., Kurniasari F.N., Sholikhati A.S., Hardianti M., Ekaputra E. (2024). Assessment of Nutritional Status in Patients with Head and Neck Cancer Before Radiotherapy: A Single-Center, Cross-Sectional Study. Medeni. Med. J..

[44] Magnano M., Mola P., Machetta G., Maffeis P., Forestiero I., Cavagna R., Artino E., Boffano P. (2015). The Nutritional Assessment of Head and Neck Cancer Patients. Eur. Arch. Oto-Rhino-Laryngol..

[45] Conlon M.A., Bird A.R. (2014). The Impact of Diet and Lifestyle on Gut Microbiota and Human Health. Nutrients.

[46] Ciernikova S., Sevcikova A., Stevurkova V., Mego M. (2023). Diet-Driven Microbiome Changes and Physical Activity in Cancer Patients. Front. Nutr..

[47] Jiang Y., Li Y. (2024). Nutrition Intervention and Microbiome Modulation in the Management of Breast Cancer. Nutrients.

[48] Ma Z., Li W. (2019). How and Why Men and Women Differ in Their Microbiomes: Medical Ecology and Network Analyses of the Microgenderome. Adv. Sci..

[49] Liu X., Tong X., Jie Z., Zhu J., Tian L., Sun Q., Ju Y., Zou L., Lu H., Qiu X. (2023). Sex Differences in the Oral Microbiome, Host Traits, and Their Causal Relationships. iScience.

[50] Pivetta G., Dottori L., Fontana F., Cingolani S., Ligato I., Dilaghi E., Milani C., Ventura M., Borro M., Esposito G. (2023). Gastric Microbiota Gender Differences in Subjects with Healthy Stomachs and Autoimmune Atrophic Gastritis. Microorganisms.

[51] Ferlay J., Soerjomataram I., Dikshit R., Eser S., Mathers C., Rebelo M., Parkin D.M., Forman D., Bray F. (2015). Cancer Incidence and Mortality Worldwide: Sources, Methods and Major Patterns in GLOBOCAN 2012. Int. J. Cancer.

[52] Peters B.A., Wu J., Pei Z., Yang L., Purdue M.P., Freedman N.D., Jacobs E.J., Gapstur S.M., Hayes R.B., Ahn J. (2017). Oral Microbiome Composition Reflects Prospective Risk for Esophageal Cancers. Cancer Res..

[53] Nearing J.T., DeClercq V., Van Limbergen J., Langille M.G.I. (2020). Assessing the Variation within the Oral Microbiome of Healthy Adults. mSphere.

[54] Yin J., Dong L., Zhao J., Wang H., Li J., Yu A., Chen W., Wei W. (2020). Composition and Consistence of the Bacterial Microbiome in Upper, Middle and Lower Esophagus before and after Lugol’s Iodine Staining in the Esophagus Cancer Screening. Scand. J. Gastroenterol..

[55] Snider E.J., Freedberg D.E., Abrams J.A. (2016). Potential Role of the Microbiome in Barrett’s Esophagus and Esophageal Adenocarcinoma. Dig. Dis. Sci..

[56] Pei Z., Bini E.J., Yang L., Zhou M., Francois F., Blaser M.J. (2004). Bacterial Biota in the Human Distal Esophagus. Proc. Natl. Acad. Sci. USA.

[57] Dong L., Yin J., Zhao J., Ma S.-R., Wang H.-R., Wang M., Chen W., Wei W.-Q. (2018). Microbial Similarity and Preference for Specific Sites in Healthy Oral Cavity and Esophagus. Front. Microbiol..

[58] Liatsos C., Papaefthymiou A., Kyriakos N., Galanopoulos M., Doulberis M., Giakoumis M., Petridou E., Mavrogiannis C., Rokkas T., Kountouras J. (2022). Helicobacter Pylori, Gastric Microbiota and Gastric Cancer Relationship: Unrolling the Tangle. World J. Gastrointest. Oncol..

[59] Li D., He R., Hou G., Ming W., Fan T., Chen L., Zhang L., Jiang W., Wang W., Lu Z. (2020). Characterization of the Esophageal Microbiota and Prediction of the Metabolic Pathways Involved in Esophageal Cancer. Front. Cell. Infect. Microbiol..

[60] Nasrollahzadeh D., Malekzadeh R., Ploner A., Shakeri R., Sotoudeh M., Fahimi S., Nasseri-Moghaddam S., Kamangar F., Abnet C.C., Winckler B. (2015). Variations of Gastric Corpus Microbiota Are Associated with Early Esophageal Squamous Cell Carcinoma and Squamous Dysplasia. Sci. Rep..

[61] Liu Y., Lin Z., Lin Y., Chen Y., Peng X., He F., Liu S., Yan S., Huang L., Lu W. (2018). Streptococcus and Prevotella Are Associated with the Prognosis of Oesophageal Squamous Cell Carcinoma. J. Med. Microbiol..

[62] Yang W., Chen C.-H., Jia M., Xing X., Gao L., Tsai H.-T., Zhang Z., Liu Z., Zeng B., Yeung S.-C.J. (2021). Tumor-Associated Microbiota in Esophageal Squamous Cell Carcinoma. Front. Cell Dev. Biol..

[63] Gao S., Li S., Ma Z., Liang S., Shan T., Zhang M., Zhu X., Zhang P., Liu G., Zhou F. (2016). Presence of Porphyromonas Gingivalis in Esophagus and Its Association with the Clinicopathological Characteristics and Survival in Patients with Esophageal Cancer. Infect. Agent. Cancer.

[64] Chen X., Winckler B., Lu M., Cheng H., Yuan Z., Yang Y., Jin L., Ye W. (2015). Oral Microbiota and Risk for Esophageal Squamous Cell Carcinoma in a High-Risk Area of China. PLoS ONE.

